# Patient‐specific dose quality assurance of single‐isocenter multiple brain metastasis stereotactic radiosurgery using PTW Octavius 4D

**DOI:** 10.1002/acm2.12979

**Published:** 2020-07-18

**Authors:** James McCulloch, Jamie Pawlowski, Neil Kirby, Karl Rasmussen, Zheng Shi, Pamela Myers, Sotirios Stathakis, Niko Papanikolaou, Daniel L. Saenz

**Affiliations:** ^1^ Department of Radiation Oncology University of Texas Health San Antonio San Antonio TX USA

**Keywords:** multiple brain metastases, patient specific, quality assurance, single isocenter, stereotactic radiosurgery

## Abstract

**Purpose:**

Single‐isocenter multiple brain metastasis stereotactic radiosurgery is an efficient treatment modality increasing in clinical practice. The need to provide accurate, patient‐specific quality assurance (QA) for these plans is met by several options. This study reviews some of these options and explores the use of the Octavius 4D as a solution for patient‐specific plan quality assurance.

**Methods:**

The Octavius 4D Modular Phantom (O4D) with the 1000 SRS array was evaluated in this study. The array consists of 977 liquid‐filled ion chambers. The center 5.5 cm × 5.5 cm area has a detector spacing of 2.5 mm. The ability of the O4D to reconstruct three‐dimensional (3D) dose was validated against a 3D gel dosimeter, ion chamber, and film measurements. After validation, 15 patients with 2–11 targets had their plans delivered to the phantom. The criteria used for the gamma calculation was 3%/1 mm. The portion of targets which were measurable by the phantom was countable. The accompanying software compiled the measured doses allowing each target to be counted from the measured dose distribution.

**Results:**

Spatial resolution was sufficient to verify the high dose distributions characteristic of SRS. Amongst the 15 patients there were 74 targets. Of the 74 targets, 61 (82%) of them were visible on the measured dose distribution. The average gamma passing rate was 99.3% (with sample standard deviation of 0.68%).

**Conclusions:**

The high resolution provided by the O4D with 1000 SRS board insert allows for very high‐resolution measurement. This high resolution in turn can allow for high gamma passing rates. The O4D with the 1000 SRS array is an acceptable method of performing quality assurance for single‐isocenter multiple brain metastasis SRS.

## 1 INTRODUCTION

Interest in stereotactic radiosurgery (SRS) to multiple brain metastases has increased in recent years due to the efficiency benefits and increased confidence in the accuracy of the technology. Several approaches are used including Varian HyperArc, BrainLab Elements, and Elekta High‐definition dynamic radiosurgery.^1^ Such an approach enables the delivery of precise stereotactic radiosurgery characterized by high doses of radiation (15–24 Gy) to multiple well‐defined small intracranial targets with a single isocenter (single setup). The elimination of multiple isocenters (one set of arcs per target) allows for treatment in a reasonable time frame without the need for extensive verification of each target. Instead one isocenter is verified, and the position of all targets relative to their planned positions is confirmed by radiographic verification of the skull bony anatomy.

Single‐isocenter multiple‐target SRS is subject to several complicating factors requiring extensive commissioning measurements, end‐to‐end testing, and patient‐specific quality assurance (QA) when intensity modulation is employed. Rotational uncertainties in the positioning of the skull or collimator angle can lead to increasing geometric misses with increasing distance of a target from the isocenter.^2^ Careful validation must be ensured before implementing this technique clinically. Furthermore, since the required dose distributions are complex, volumetric‐modulated arc therapy (VMAT) is employed. This requires patient‐specific dose validation, as is recommended by various entities.^3^, ^4^

Patient‐specific dose validation is typically performed by recalculating the planned dose onto a phantom in which a measurement is then made.^4^ Measurement and calculation are compared with a formalism such as gamma analysis described by Dan Low et al.^5^ When implementing this approach for single‐isocenter multiple‐target SRS, however, extra factors are at play. First, a large fraction of the three‐dimensional (3D) dose distribution is in regions of very low dose (as is desired for normal brain sparing). Attention must be paid to ensure the analysis is not dominated by low dose regions, thereby masking any disagreement in the small fraction of the dose distribution in the brain metastases. Second, it is quite difficult to perform 3D dosimetry with conventional methods and phantoms commonly used for patient‐specific dose quality assurance. However, gel dosimetry or a solid polyurethane‐based dosimeter (e.g., PRESAGE™) can be used to measure a 3D dose distribution.^6^, ^7^ Various two‐dimensional (2D) methods can be used to acquire representative slices in a 3D dose distribution, but representative slices are very difficult to identify for multiple brain metastasis SRS since any plane will only capture a subset of brain metastases. Third, since the regions of high dose are quite small, small‐field dosimetry concerns are heightened, and only appropriate detectors are indicated for this type of measurement.^8^

There are multiple patient‐specific QA techniques which could be applied to single‐isocenter multiple‐target SRS. The film and ionization chamber measurement method employs a film placed at a depth in a phantom to acquire a relative dose distribution as well as an ionization chamber for absolute dose assessment. For the calculation, the treatment plan is recalculated on the phantom geometry often with the beam angles modified (e.g., table angles collapsed to one angle or static beams delivered all perpendicular to the film plane). This method allows for dose to be verified, but focuses only on one (or several, if possible) planes. Positioning of both the ionization chamber and film is crucial to obtaining a meaningful measurement. For example, the ionization chamber should be placed in a target in order to record a meaningful dose. The position of targets relative to the isocenter is variable for each plan, and thus requires different ionization chamber placement in each plan, complicating the process. It is often only possible to place film in a sagittal, axial, or coronal orientation thereby limiting the possible 2D plane positions increasing the difficulty of capturing dose to multiple targets.

Detector arrays cast in various 2D and 3D dimensions are also widespread in use and can facilitate the patient‐specific QA process. The Scandidos (Uppsala, Sweden) Delta4 Phantom+ is a PMMA or plastic water phantom with two diode detector boards perpendicular to each other. The distance between the detectors is 5 mm in the inner 6 cm × 6 cm area and 10 mm in the outer 20 cm × 20 cm area.^9^ The Delta4 does allow for two consecutive measurements to be compiled. The second measurement can increase resolution by shifting the phantom 2.5 mm in the longitudinal direction. This allows for much higher resolution when comparing the measured plan to the calculated plan. For radiosurgery plans, the phantom would need to be shifted in order to acquire higher resolution. Since the diode arrays are static, additional uncertainty occurs for control points in which the beam is oriented directly parallel to one of the boards. Moreover, in single‐isocenter multiple‐target SRS, high dose distributions will be delivered to arbitrary compact volumes, very few of which would coincide with any of the detector boards. This is quite different than the typical use of the Delta4 Phantom+ for patient‐specific quality assurance measurement where the delivered dose is situated at or near the isocenter, where the two bisecting detector board plans will generally intersect large portions of the 3D dose distribution.

The Sun Nuclear (Melbourne, FL) ArcCHECK QA phantom is composed of 1386 diode detectors in a cylindrical shape. ArcCHECK has an insert that can be inlayed into the phantom for an ionization chamber.^10^ An ionization chamber inside of the phantom could allow for absolute dose assessment. The detectors are spaced 1 cm apart. The 1 cm detector distance could be detrimental for SRS QA.^11^ Metastasis is often on the order of millimeters. Due to the steep dose gradients, the large detector spacing could prevent the dose profile near a metastasis from being measured to a degree sufficient to verify dose delivery accuracy.

The Sun Nuclear SRS MapCHECK is a planar detector array consisting of 1013 diodes. The array has a measurable area of 77 mm × 77 mm.^12^ The SRS MapCHECK can be inserted into the Sun Nuclear StereoPHAN which holds the SRS MapCHECK over the superior end of the couch. The StereoPHAN is a cylindrical PMMA phantom which is shaped in order to mimic the shape of a head.^13^ The StereoPHAN holds the detector board in place. The detector does not rotate with the gantry. The software that accompanies this product allows for beams to be delivered to the phantom at noncoplanar couch angles up to ±45°.

Log files can also be used to determine the position of individual MLCs during treatment. The MLC actual position can be compared to their planned position in order to create a delivered fluence which can be compared with the planned fluence. Studies have shown that there is a high correlation between these log file tests and ionization chamber array measurements.^14^ However, no radiation is measured directly in the patient or phantom geometry. By only measuring the MLC positions, possible dosimetric errors may be overlooked. Particularly, when commissioning a new program as complex as single‐isocenter multiple‐target SRS, direct measurements will be initially preferable.

Electronic portal imaging device (EPID) dosimetry is another choice for patient‐specific QA. The EPID is extended and the plan is delivered to it during image acquisition in cine mode. These images are used to determine the leaf position at each control point. Planned leaf position can then be compared to actual leaf position. A revised gamma calculation can be used to determine if the plan is passing or not.^15^ Dose is not measured with this method allowing for possible errors to occur.

In this study, we describe our experience commissioning and using the PTW (Freiburg, Germany) Octavius 4D Modular Phantom (O4D) with the Octavius 1000SRS array to conduct patient‐specific dose quality assurance for single‐isocenter multiple‐target SRS patients planned using BrainLab Elements. The linear accelerator was the Novalis TX equipped with a 6‐MV SRS beam energy with a light flattening‐filter capable of delivering dose rates of 1000 MU/min. We will describe the specific advantages of using this platform for measurements and describe the limitations as well. The initial validation of the phantom is described as along with the results from a subset of the first treated patients. Some recommendations will also be presented.

## 2 MATERIALS AND METHODS

### 2.A Phantom

**Fig. 1:**
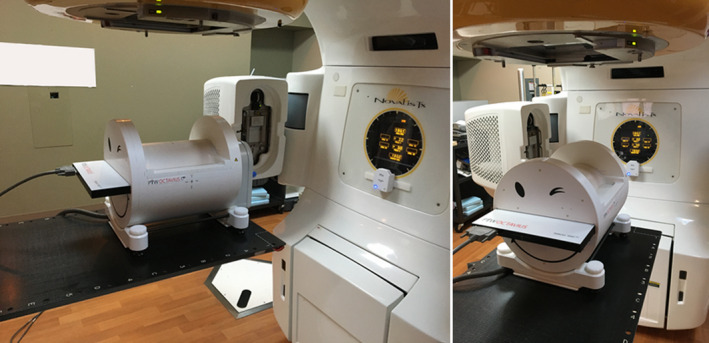
The Octavius four‐dimensional Modular Phantom setup for stereotactic radiosurgery patient‐specific quality assurance.

The Octavius 4D Modular Phantom (O4D) is a cylindrical phantom consisting of a water equivalent plastic (1.05 g/cm^3^). The phantom has an insert in the center for a detector board. This study employed the 1000 SRS board insert with the OCTAVIUS top SRS (which defines the radius of the cylindrical measurement) creating an effective phantom diameter of 17 cm, approximating that of a typical patient head size. An inclinometer was placed on the gantry to detect gantry angle. The components of the phantom then rotate with the gantry synchronously in order to maintain positioning such that the detector board is perpendicular to the radiation beam. The phantom and inclinometer can both be seen in Fig. 1. The O4D is comprised of many moving parts requiring consistent performance, but studies have demonstrated the robustness and accuracy of the phantom.^16^

At the time of commissioning, the MU setting for a 5 × 5 cm^2^ field to deliver 2 Gy with 6 MV to the phantom isocenter was determined. The same number of monitor units for the corresponding energy is then delivered each day to the phantom prior to use. This procedure “… inherently corrects for the temperature, pressure, and energy dependence and eliminates any deviation linked to the daily machine output fluctuation”.^17^ After the daily correction is complete, patient quality assurance plans are subsequently delivered.

The software tool for measurement and analysis is PTW Verisoft. Verisoft compiles data from multiple measurements and conducts 4D dose reconstruction in coplanar and noncoplanar geometries. Raw dose profiles measured on the detector array are postprocessed in Verisoft to build a 3D dose distribution. The process by which this is performed is completed in the commissioning process and consists of providing measured percent depth dose curves at an SSD of 85 cm for field sizes ranging from 4 × 4 cm^2^ to 26 × 26 cm^2^ and also providing the electron density of the phantom relative to water. The electron density is an editable value since it is recommended by PTW to adjust this parameter slightly to obtain agreement between measurement and calculation for simple square fields in the commissioning process.

An advantage of the O4D with the 1000 SRS board insert is the high resolution it provides. The board consists of 977 liquid‐filled ion chambers.^18^ The detectors have a volume of 0.0003 cm^3^. The center 5.5 cm × 5.5 cm area has a detector spacing of 2.5 mm. The rest of the array out to the 11 cm × 11 cm edges has a detector spacing of 5 mm. The small detector spacing allows for increased resolution. The higher resolution can result in higher accuracy calculations.

The detector size is consistent throughout the board. The detectors in the outer periphery are the same as those on the interior. The Verisoft gamma calculation allows for interpolation between points. If a reference dose point has no measured dose point near it, then Verisoft uses the method described by Depuydt et al.^19^ to interpolate between points. In this way the greater detector spacing on the periphery should have a reduced effect on the gamma analysis.

To justify the accuracy of the O4D phantom with the 1000 SRS board, a validation study was done alongside a gel dosimeter measurement which was done for initial commissioning of the single‐isocenter multiple‐target SRS program. A patient with eight intracranial brain metastases was planned using BrainLab (Munich, Germany) Elements Multiple Brain Metastasis version 2.0 treatment planning software. All lesions were treated to 8 Gy in preparation for gel dosimetry using the RTsafe (Athens, Greece) PsuedoPatient gel phantom. The plan was delivered both to the gel phantom, a 3D printed head phantom filled with dosimetric gel machined to match the bony anatomy of the patient with submillimeter accuracy, as well as to the O4D phantom. Delivering the same plan to both phantoms and assessing the agreement of each measured dose with the treatment planning calculation allows for end‐to‐end verification of the O4D to reconstruct 3D measured dose distributions. The gel phantom results were compared to the calculation by RTsafe remote dosimetry program, including 3D gamma analysis with 3%/2 mm criteria due to the 2 mm MRI slice thickness, dose and DVH statistics, and isodose line comparison.

After initial validation of the O4D, two control plans (dosimetric and localization) were devised on the Standard Imaging (Middleton, WI) Lucy phantom to characterize the baseline performance of the O4D for subsequent troubleshooting should the need arise. The dosimetric plan and subsequent measurement also help to validate absolute dose accuracy by measurement with both the O4D and a Standard Imaging A16 ionization chamber. The A16 ionization chamber is cross‐calibrated with an ADCL‐calibrated PTW 31013 ionization chamber on an annual basis for an estimation of absolute dose. The dosimetric standard plan consisted of a single target of a 3‐cm diameter treated to 8 Gy. The localization plan consisted of four targets, each situated at the positions of four markers in the film cassette which each make an impression on the radiochromic film upon closing the cassette. Localization accuracy was assessed by scanning the irradiated film with an EPSON Perfection V750 PRO scanner (Seiko Epson, Japan) and measuring the difference between the cloud of radiation and the film markers using RIT software (Radiological Imaging Technology, Inc., Colorado Springs, CO).

### 2.B Patient study design

The BrainLab Elements Multiple Brain Metastasis version 2.0 treatment planning software was used in this study to evaluate the O4D’s ability to perform SRS quality assurance. The treatment planning system uses a single isocenter to treat multiple brain metastases and optimizes MLC apertures to obtain optimal conformity index (CI) and gradient index (GI). For this study, CI was defined as the prescription isodose volume divided by the PTV volume. GI was defined as the 50% isodose volume divided by the PTV volume. The number of targets varied from 2 to 11. The planning system employs both clinical protocols (prescription dose, normal tissue tolerances, etc.) and geometric protocols (beam geometries) to automate the planning workflow. Noncoplanar arcs are used to create the most conformal dose distributions using five different couch angles to provide different angles of dose deposition. The plan is calculated with a dose grid of 1 mm. The isocenter position is determined as the average position of the centers of mass of each PTV. This type of centroid placement is called a point centroid.^20^ The point centroid technique is independent of the volume and places the isocenter in a representative position of the targets thereby having the effect of maximizing the amount of targets which are measurable in a quality assurance plan.

The first 15 clinically treated radiosurgery plans were evaluated in this study (Table 1). Each patient plan was recalculated on the O4D phantom with a dose grid of 1 mm. Noncoplanar arc angles were used in the QA plan calculation (even though measurement is performed at a table angle of 0°). This is done because the O4D phantom rotates about the axis of the treatment table. At a table angle of 0 degrees, the O4D rotates the detector board so that it is always perpendicular to the rotating gantry. At a noncoplanar angle, the O4D would continue to rotate but it would not be perpendicular to the gantry. While the plans are delivered to the O4D at a couch angle of 0°, Verisoft allows for the dose to be compiled into a noncoplanar dose reconstruction. The user specifies the noncoplanar angle at which each beam will be delivered to the patient. It then reconstructs the measured dose to be at that noncoplanar angle. Verisoft produces a measured dose distribution that includes the noncoplanar angles. This allows for the measured dose profiles to reflect the true position of the lesions relative to the isocenter. Each treatment plan was delivered to the phantom over several sessions.

For the analysis, the gamma index was calculated by the software using the method described by Dan Low et al.^5^ The criteria used for the gamma calculation was 3%/1 mm. Task Group 218 recommends a general 3%/2 mm criteria for IMRT QA (10% dose threshold) relative to a global normalization to maximum dose and recommends tighter tolerances for SRS/SBRT.^4^ This study employed the 3%/1 mm due to the need for high geometric agreement and used normalization to maximum dose with a 10% dose threshold as recommended. A minimum percentage of 90% or more points passing the gamma criteria is required for an acceptable plan.

A limitation of the O4D with the 1000 SRS board insert is that the detector array is only 11 cm × 11 cm. This does not always allow for every target to be measured. The fraction of metastases which are measurable by the O4D was directly countable from the dose profiles. Targets most likely to be outside the measurable volume are those farthest from isocenter and therefore the most sensitive to rotational uncertainties in setup or MLC errors as shown by Roper et al.^21^ This was another factor motivating the need for the gel dosimetry validation prior to clinical use in order to validate the measured dose to peripheral targets.

### 2.C Error detectability analysis

Gamma analysis is an effective tool to quantitatively compare dose distributions but can overlook errors particularly when high pass rates are observed. Hence, to investigate the robustness of these measurements and the detectability of small errors, the gamma results were recalculated with a 1%/1 mm criteria. To further test the robustness of the system, errors were artificially introduced. First, the measured dose was scaled by 3% with a scaling f factor in Verisoft. Another test of the system was shifting the measured dose in the lateral direction by 1 mm. The resulting recalculated gamma passing rates were recorded for 3%/1 mm criteria as well as 1%/1 mm criteria.

**Table 1:** Patient demographics.

Patient number	Number of targets	Average CI	Average GI	Prescription volume
1	5	1.31	3.88	99.5%
2	2	1.21	2.93	99.5%
3	5	1.37	5.55	99.5%
4	4	1.55	4.12	99.5%
5	3	1.31	3.25	99.5%
6	4	1.71	3.93	99.5%
7	11	1.60	6.10	99.5%
8	10	1.63	6.01	99.5%
9	3	1.31	4.47	99.5%
10	4	1.39	4.97	99.5%
11	5	1.32	5.32	99.5%
12	9	1.61	6.89	99.5%
13	3	1.47	4.75	99.5%
14	3	1.33	3.54	99.5%
15	3	1.53	6.53	99.5%

## 3 RESULTS

**Fig. 2:**
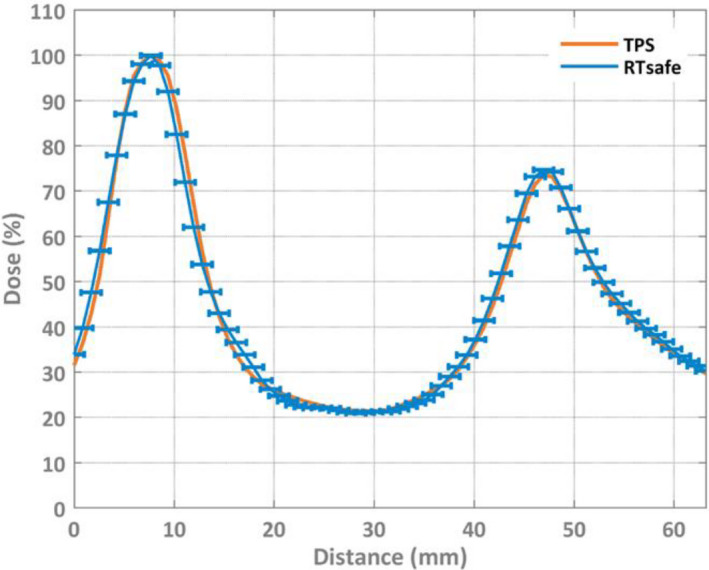
Sample one‐dimensional dose profile showing both the measurement (RTsafe) and the calculation (TPS). 1 mm positional bars are shown to indicate the generally accepted geometrical tolerance of stereotactic radiosurgery compared with the distance between the curves.

The results from the validation study indicated agreement between the gel measurement and the patient plan, as assessed by RTsafe remote dosimetry 3D gamma analysis, as well as agreement between the O4D measurement and phantom recalculation, as assessed by 3D gamma analysis in Verisoft. The gel dosimetry analysis was performed in each target, and the mean gamma passing rate across all targets was 97.8% (3%/2 mm) with a sample standard deviation of 2.3% (range 94.5%–100%). The results of the O4D measurement were an overall gamma passing rate of 99.9% (3%/2 mm). When tightening the O4D gamma criteria to 3%/1 mm to match that used the patient study, a passing rate of 99.3% was obtained. In the validation study, the slightly better agreement of the O4D measurement with calculation as compared to the gel dosimeter measurement with calculation was attributed to fewer uncertainties inherent in the process than for gel dosimetry. A sample 1D dose profile from the gel dosimeter is shown in Fig. 2.

**Fig. 3:**
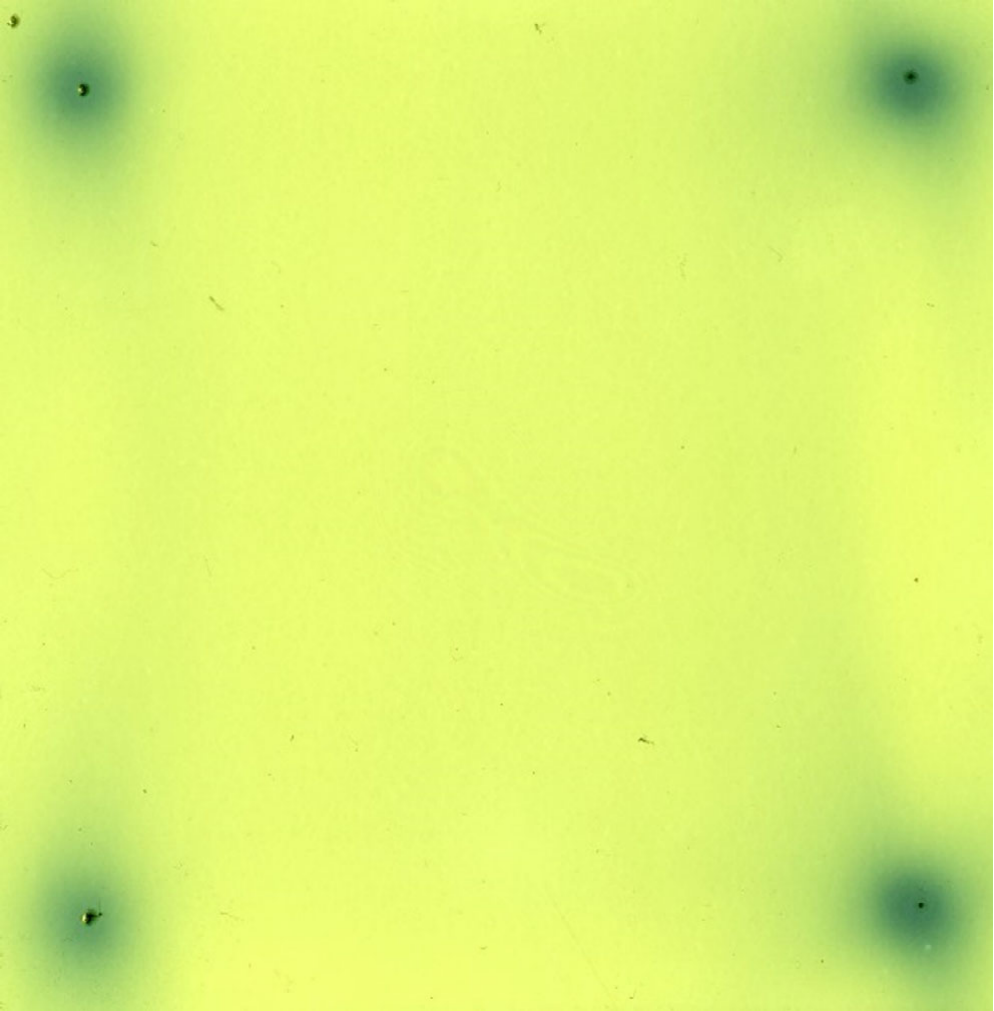
Scan of the radiochromic film with localization plan indicating the dose clouds for the four targets as well as the planned dose cloud positions indicated by the small opaque markers.

Regarding the dosimetric and localization control plans, the gamma passing rates (3%/1 mm) were 99.9% and 94.6% respectively. Absolute dose was measured as 972 cGy as compared to the anticipated 989 cGy calculated from the treatment plan, a difference of 1.7%. In the localization plan, the offset of the radiation cloud from the center of the impressions on the film differed by no more than 0.6 mm. An image of the scanned film is shown in Fig. 3.

**Fig. 4:**
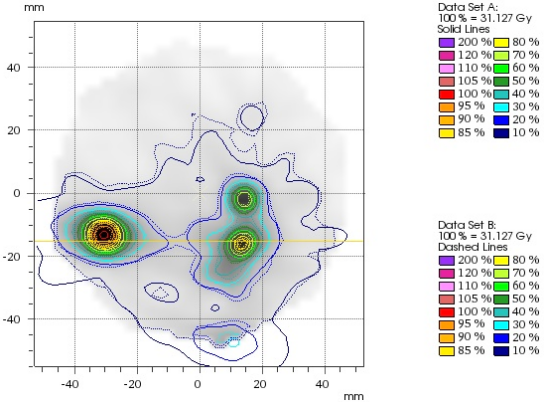
Measured Dose from a single slice of the O4D phantom. The Dash lines show the calculated plan and the solid lines show the measured plan.

**Fig. 5:**
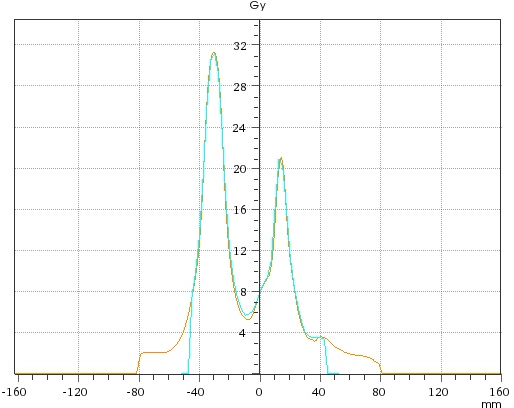
Measured dose profile (blue) versus calculated dose profile (orange).

For the patient study, high resolution dose distributions representing the position of all targets at all table angles were recorded and are exemplified in Figs. 4 and 5. Figure 4 shows a sample 2D plane with measurement isodose distributions. Figure 5 illustrates a sample profile (measurement vs. calculation) at the indicated profile position in Fig. 4.

The gamma passing rates at 3%/1 mm and at 1%/1 mm are shown in Table 2 in addition to the total number of targets and measurable targets per patient. Table 3 presents overall statistics across all patients.

**Table 2:** Individual results for each patient.

Patient number	Number of targets	Number of measurable lesions	Gamma passing rate (3%/1 mm)	Gamma passing rate (1%/1 mm)
1	5	4	99.4%	65.1%
2	2	2	100%	97.7%
3	5	3	99.4%	91.9%
4	4	4	99.8%	88.1%
5	3	1	99.9%	94.9%
6	4	3	99.7%	86.7%
7	11	8	98.9%	63.0%
8	10	9	99.4%	69.3%
9	3	3	99.5%	95.6%
10	4	3	98.0%	87.5%
11	5	5	98.2%	88.3%
12	9	8	98.1%	83.9%
13	3	3	100%	100%
14	3	3	99.5%	96.9%
15	3	2	99.7%	91.4%

**Table 3:** Summary of results.

Total number of targets	74
Total number of measurable targets	61
Average gamma passing rate (3%/1 mm)	99.3%

The 15 patients in the study had 74 targets amongst them. Of these 74 targets, 61 were visible in the dose profiles. (82.4%). These targets were considered measurable. Of the measurable targets, all of them had gamma scores greater than or equal to 98%. A majority of the plans had a score >99%. The average gamma score was 99.3%.

When introducing artificially produced errors of 3% positive dose scaling and a 1‐mm lateral shift, the gamma results were recorded with a 3%/1 mm tolerance and a 1%/1 mm tolerance. The results of this can be seen in Table 4. The average 3%/1 mm gamma passing rate is high with a small standard deviation. Using the same criteria to evaluate, the average gamma score decreases both for the 1‐mm shift in the lateral direction for a scaled dose of 3%. Each of these average scores is well above the universal action limit of 90% recommended by Task Group 218.^4^ Using a gamma analysis with a 1%/1 mm criteria results in an average score of 86.7% and the average scores for a shifted dose or a scaled dose are markedly different from the original average gamma score. These results reveal that the Octavius 4D system can detect small errors when simultaneously examining tighter gamma criteria.

**Table 4:** Gamma Passing rates for the measured data, data that has been shifted in the LR direction, and data that has been scaled by 3%.

	Gamma passing rate (3%/1 mm)			Gamma passing rate (1%/1 mm)		
	Original	1 mm lateral Shift	3% positive dose scaling	Original	1 mm lateral Shift	3% positive dose scaling
	99.4	96.2	98.6	65.1	62.1	44.9
	100	97.1	94.4	97.7	87.4	74.6
	99.4	96.6	99.3	91.9	83.7	84.2
	99.8	98.6	98.2	88.1	77.8	64
	99.9	98.9	98.9	94.9	83.7	80.6
	99.7	98.3	99.3	86.7	81.7	67.6
	98.9	97.7	97.2	63	59.9	38.7
	99.4	96.8	98.2	69.3	60.7	46.3
	99.5	98	99.4	95.6	90.6	91.6
	98	95	97.3	87.5	78.8	79.4
	98.2	94.5	90.1	88.3	81.2	60.5
	98.1	92.6	97.8	83.9	67.7	80.7
	100	98.9	99.9	100	91.9	99.2
	99.5	95.1	99.6	96.9	82.7	98.2
	99.7	97.3	97.8	91.4	81	77
Average	99.3	96.8	97.7	86.7	78.1	72.5
Standard deviation	0.68	1.82	2.51	11.78	10.51	18.73

## 4 DISCUSSION

The validation study, while showing better agreement for the O4D than gel dosimetry, nevertheless revealed a high degree of agreement in both systems. The agreement with both systems with calculation justifies using the O4D to approximate the 3D dose since it was shown that O4D agrees with calculation as well as a true 3D dosimeter.

The standard plans proved valuable to establishing baseline dosimetric measurements to be compared with longitudinal O4D measurements. The localization plan delivered to the O4D phantom can now be remeasured at any later date and the dose distribution compared with that established at the time of initial validation alongside film measurement for any changes in localization accuracy.

Visual inspection of the dose profiles proves difficult since only 2D profiles within a 3D space are easily compared. Since regions of high doses are small and isolated, visual inspection requires searching for these regions to visualize relevant dose profiles to compare between calculation and measurement. Gamma analysis, on the other hand, does provide a vital quantitative overall assessment. However, only a small fraction of the 3D volume is in high dose regions and requires revisiting the low dose threshold used in analysis. We only suppressed doses lower than 10% of the maximum calculated dose, but other institutions may wish to assess agreement at the higher dose levels as well, at least at the time of program commissioning.

Approximately 80% of lesions were close enough to isocenter to be measurable. This percentage was compared to expectation making basic assumptions about the typical brain dimensions. The average brain size of a “… well‐nourished population of predominantly European ancestry is about 1260 cc for men and 1,130 for women”.^22^ The cylinder has a radius of 5.5 cm and a height of 11 cm which gives a volume of 1045 cc. This cylinder covers the entire active area of the board. In a best‐case scenario where the volume this cylinder covers is completely the brain tissue, the phantom would cover 83% of a male brain and 92% of the female brain. Assuming the patients in this study have average brain sizes and are of European descent, it can be expected that 83%–92% of the brain will be covered.

This study resulted in 82% of the targets covered. This is very close to the lower bound of 83% which comes from the aforementioned brain size study. One explanation for this might be that the study only had 15 data points and that was not enough to conform to the standard brain size population. It is also possible that the patients were not of European descent, which would cause the quoted study to not apply to them. BrainLab Elements also place the isocenter at the centroid of the cluster of targets, thereby minimizing the distance to all targets which could also contribute to a slightly better measurable coverage of targets.

The Verisoft software that accompanies the O4D allows for the user to measure a plan at one couch location, then shift the direction of the couch in one of the three cardinal directions, redeliver the same plan, and compile the two plans together. This is an option to effectively create a larger effective measuring volume. However, this technique is not compatible with noncoplanar dose reconstruction and was therefore not employed in this study. It does remain an option for plans with peripheral lesions of particular concern.

Furthermore, PTW will soon be releasing a 16 cm × 16 cm SRS array that will be able to be used in the same fashion as the 1000 SRS board was used in this study. A larger array in the O4D phantom would cover a larger volume and allow for more targets to be measured. With a similar detector spacing with the 1000 SRS, we would expect similar performance with better measurable coverage.

Uncertainty is inherent in this process in the measurements. To estimate the uncertainty of the gamma passing rate, a plan was remeasured on the O4D five additional times. Patient 11 was chosen for this purpose as a conservative assumption due to its lower passing rate, potentially making it possible to observe more intermeasurement variability than a nearly 100% passing quality assurance plan. Acquiring more than five measurements would be ideal, but is prohibitively difficult in practice due to practicality constraints. Of the five remeasured plans, the sample standard deviation in the gamma passing rate was 0.4%. The gamma passing rate is not likely normally distributed given the natural limit of 100% maximum passing rate. However, it represents a small uncertainty in comparison with the difference between the mean passing rate of 99.3% and the minimum passing rate of 90.0%.

## 5 CONCLUSIONS

The high resolution provided by the O4D with 1000 SRS board insert allows for very high‐resolution measurement. This high resolution in turn can allow for gamma analysis with a stringent 1 mm distance‐to‐agreement. An issue this phantom has is the limited region covered by the detector board. However, the board covers most cranial targets in this study and will be mitigated by an upcoming 16 × 16 cm^2^ array. The O4D with 1000 SRS board is an acceptable method of performing quality assurance for multiple brain metastasis SRS plans.

## CONFLICT OF INTEREST

This work was supported in part by a grant from Brainlab.
